# Peritoneal Carcinosis: What the Radiologist Needs to Know

**DOI:** 10.3390/diagnostics13111974

**Published:** 2023-06-05

**Authors:** Alfonso Reginelli, Giuliana Giacobbe, Maria Teresa Del Canto, Marina Alessandrella, Giovanni Balestrucci, Fabrizio Urraro, Gaetano Maria Russo, Luigi Gallo, Ginevra Danti, Barbara Frittoli, Luca Stoppino, Daria Schettini, Franco Iafrate, Salvatore Cappabianca, Andrea Laghi, Roberto Grassi, Luca Brunese, Antonio Barile, Vittorio Miele

**Affiliations:** 1 Department of Precision Medicine, University of Campania “L. Vanvitelli”, 80138 Naples, Italy; 2 General and Emergency Radiology Department, “Antonio Cardarelli” Hospital, 80131 Naples, Italy; 3 Department of Radiology, Azienda Ospedaliero-Universitaria Careggi, 50134 Florence, Italy; 4 Department of Radiology, Spedali Civili Hospital, 25123 Brescia, Italy; 5 Department of Radiology, University Hospital of Foggia, 71122 Foggia, Italy; 6 Department of Radiology, Villa Scassi Hospital, Corso Scassi 1, 16121 Genova, Italy; 7 Department of Radiological, Oncological and Pathological Sciences, Policlinico Umberto I, Sapienza University of Rome, Viale Regina Elena 324, 00161 Rome, Italy; 8 Department of Medical Surgical Sciences and Translational Medicine, Sapienza—University of Rome, Radiology Unit—Sant’Andrea University Hospital, 00189 Rome, Italy; 9 Department of Medicine and Health Sciences “Vincenzo Tiberio”, University of Molise, 86100 Campobasso, Italy; 10 Department of Applied Clinical Sciences and Biotechnology, University of L’Aquila, 67100 L’Aquila, Italy; 11 Department of Translational Research, Diagnostic and Interventional Radiology, University of Pisa, 56126 Pisa, Italy

**Keywords:** peritoneal carcinosis, peritoneal cancer index, computed tomography, ultrasound, magnetic resonance imaging, radiomic features

## Abstract

Peritoneal carcinosis is a condition characterized by the spread of cancer cells to the peritoneum, which is the thin membrane that lines the abdominal cavity. It is a serious condition that can result from many different types of cancer, including ovarian, colon, stomach, pancreatic, and appendix cancer. The diagnosis and quantification of lesions in peritoneal carcinosis are critical in the management of patients with the condition, and imaging plays a central role in this process. Radiologists play a vital role in the multidisciplinary management of patients with peritoneal carcinosis. They need to have a thorough understanding of the pathophysiology of the condition, the underlying neoplasms, and the typical imaging findings. In addition, they need to be aware of the differential diagnoses and the advantages and disadvantages of the various imaging methods available. Imaging plays a central role in the diagnosis and quantification of lesions, and radiologists play a critical role in this process. Ultrasound, computed tomography, magnetic resonance, and PET/CT scans are used to diagnose peritoneal carcinosis. Each imaging procedure has advantages and disadvantages, and particular imaging techniques are recommended based on patient conditions. Our aim is to provide knowledge to radiologists regarding appropriate techniques, imaging findings, differential diagnoses, and treatment options. With the advent of AI in oncology, the future of precision medicine appears promising, and the interconnection between structured reporting and AI is likely to improve diagnostic accuracy and treatment outcomes for patients with peritoneal carcinosis.

## 1. Background 

Peritoneal carcinomatosis (PC) is defined as the insemination and implantation of neoplastic cells in the peritoneal cavity and represents an advanced stage of tumors, mostly those that develop in the abdominal –pelvic organs [1,2]. 

Therefore, PC represents a common metastatic location, especially for carcinomas of the gastrointestinal tract and ovaries [3,4,5,6]. In fact, according to the literature, approximately 75%, 17%, and 10% of patients with ovarian, gastric, and colorectal cancer, respectively, show peritoneal metastases (PM) at the time of clinical diagnosis [7,8].

The condition is generally associated with a poor prognosis, with an average survival of approximately six months (range 1–9 months) from initial diagnosis [9].

However, five-year survival rates of up to 50% are reported in well-selected groups of patients with metastatic ovarian and colorectal cancer [10,11,12].

In recent years, in fact, more aggressive loco-regional treatment strategies, such as cyto-reductive surgery (CRS), followed by hyperthermic intraperitoneal chemotherapy (HIPEC), have shown promising results for patients with limited, resectable peritoneal disease [12,13,14].

These multidisciplinary therapies are based on the concept of the peritoneum as an organ, and it is hypothesized that better prognoses are achieved with the complete removal of disease from the peritoneum [15]. 

However, the optimal selection of patients with peritoneal metastases who may benefit from curative surgical treatment remains critical [16,17].

An important problem for the treatment of PM remains, in fact, the early identification of carcinosis sites, in order to select patients to undergo cyto-reductive surgery (CRS) and hyperthermic intraperitoneal chemotherapy (HIPEC) [18,19].

A commonly applied regimen for quantifying the extent of PC is the Peritoneal Cancer Index (“PCI”) developed by Sugarbaker, which involves the division of the abdominal cavity into nine quadrants (R0: central abdomen; R1: right upper; R2: epigastrium; R3: left upper; R4: left flank; R5: left lower; R6: pelvis; R7: right lower; R8: right flank) and four intestinal segments (R9: upper jejunum; R10: lower jejunum; R11: upper ileum; R12: lower ileum) [20,21]. 

The PCI is a semi-quantitative indicator for determining the extent of peritoneal tumor spread. The success of complete cytoreductive surgery and patient prognoses are related to PCI [22]. 

Currently, exploratory laparoscopy still represents the gold standard for quantifying peritoneal disease. This is an invasive procedure, however, which is often challenging and incomplete due to adhesions and carries a small risk of complications. This underscores the need for a robust imaging modality to reliably quantify the extent of peritoneal disease [23]. 

Regarding the role of imaging, multidetector computed tomography (CT) with the intravenous administration of contrast agent is the standard method in the evaluation of PC. Magnetic-resonance imaging (MRI) can provide complementary data. Positron emission tomography (PET), on the other hand, has a more limited role: its main a is the detection of extraperitoneal involvement in cases of non-mucinous neoplasms [24,25].

Precise diagnoses based on imaging findings alone, however, are often not possible: CT findings are, in fact, often nonspecific, and a number of non-neoplastic and benign neoplastic conditions of the peritoneum may mimic malignant conditions [26]. 

Nevertheless, imaging characteristics combined with relevant clinical and demographic patient data can help narrow the field.

The purpose of this article is, therefore, to report the role of imaging in the diagnosis of peritoneal carcinosis and in the selection of patients who can benefit from surgical treatment, as well as to illustrate the typical imaging findings that every radiologist should know for this purpose.

## 2. What Radiologists Should Know about Imaging Techniques

### 2.1. Ultrasound

Abdominopelvic ultrasound (US) is the basic technique used for patients with a clinical suspicion of peritoneal carcinomatosis. In fact, US is harmless to the patient as no radiation is emitted, it easily detects peritoneal fluid, and it can also identify peritoneal implants, although the careful evaluation of all the peritoneal structures can be time consuming. However, as is well known, it is an operator-dependent technique [25,27,28].

**Figure 1:**
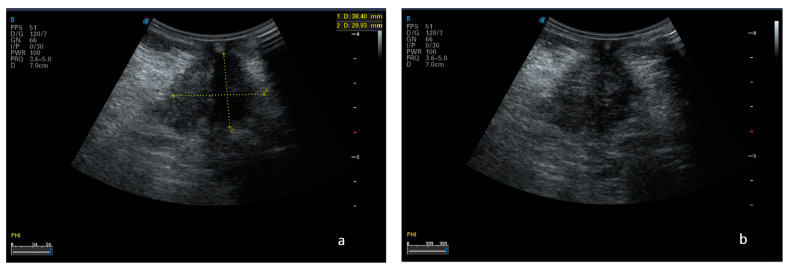
Ultrasound scan (**a**,**b**) of an 81-year-old man’s abdomen in supine position reveals a 3.8 × 2.9-cm hypoechoic lesion (nodule of peritoneal carcinosis) on the middle abdominal wall.

Initially, an US investigation of the peritoneum and peritoneal cavity should be performed with a low-frequency transducer (3.5-MHz or 5-MHz probe) to allow the evaluation of all the abdominopelvic contents, including solid organs. After the initial evaluation, a high-frequency probe can be used to visualize in more detail/precision any lesions found. In female patients, transvaginal ultrasound should also be used to evaluate the peritoneal cavity, which is a common site of peritoneal disease, and the pelvic organs (Figure 1) [29].

Furthermore, US also allows the accurate assessment of ascites volume [30,31]. 

In addition, US is not only much more sensitive than CT in providing quantitative information on the presence of even minimal amounts of free intraperitoneal fluid, but it can also provide qualitative information on the benign or malignant nature of ascites itself [32].

In fact, malignant ascites, an indirect sign of CP, often appears corpuscular and septate because of its high protein content. In contrast, the findings of nodules, sheet-like tumor masses, and/or a combination of the two are direct signs of PM [33].

In addition, when there is a substantial amount of ascites, the parietal peritoneum is seen more clearly as a regular hyperechogenic line than in anechogenic ascites; in this case, even a small 2–3-mm nodule may be visualized in the presence of ascites [34,35]. 

Thickening of the mesentery, omentum, and possible adhesions between the loops of the small bowel and peritoneal masses, as well as impaired peristalsis, are other findings that may be noted on ultrasound examination [36,37]. 

Ultrasonographic imaging also plays a key role in the identification of tumor deposits in the periumbilical region (Sister Mary Joseph nodules), which may sometimes represent the only visible manifestation of a disseminated intra-abdominal neoplasm. In these cases, a solid hypoechogenic lobulated superficial peri-umbilical mass is evident, with a partly cystic structure and internal vascularity on color Doppler. It has been demonstrated, in fact, that CP metastases can reach the umbilicus through direct extension from the anterior peritoneum or through embryonic remnants, such as the sickle-cell ligament, the median umbilicus, and the onphalo-mesenteric ligaments, through hematogenous spread, retrograde lymphatic flow, or, finally, through implant entrapment along the laparoscopic entry point [38]. 

Therefore, an accurate preoperative assessment of tumor extension is crucial for the adequate estimation of the risks and benefits associated with aggressive surgical procedures, which are often necessary in order to obtain complete cytoreduction.

Although ultrasound investigation, performed by an experienced operator, can play a central role in the primary diagnosis of gynecologic malignancies, in the assessment of the extension of tumors into the pelvis and abdominal cavity, to date, few data are available in the literature. In this regard, Zhenhong Qi et al. demonstrated that US has high accuracy in staging patients with advanced ovarian cancer [39]. 

In contrast, according to Lei Liang et al., ultrasound has potential value in preoperative PCI evaluations, the diagnosis of PM, and the differentiation of cystic and solid lesions. In this study, the preoperative ultrasound assessment of PCI was compared with the surgical assessment of PCI to explore the value of applying ultrasound in the preoperative assessment of PCI. The results showed that preoperative ultrasound can predict PCI, particularly in regions 0–7. In particular, the total score correlations in regions 0–3 and 6 were the highest. The lesions in the large omentum were easily detected by ultrasound, and the predictive value of preoperative ultrasound was good. However, ultrasound did not visualize regions 9–12, which include the superior jejunum, inferior jejunum, and the superior and inferior ileum. Therefore, these regions were excluded from the calculation of ultrasound PCI and surgical PCI in order to avoid bias in the quantification of tumor burden [40,41]. 

Finally, ultrasound makes it possible to guide the fine-needle cytological aspiration (FNAC) of ascitic fluid of undetermined origin and the US-guided biopsy of the omentum or of an accessible intra-peritoneal lesion, providing adequate samples for histological diagnoses [42,43].

### 2.2. Computed Tomography

**Figure 2:**
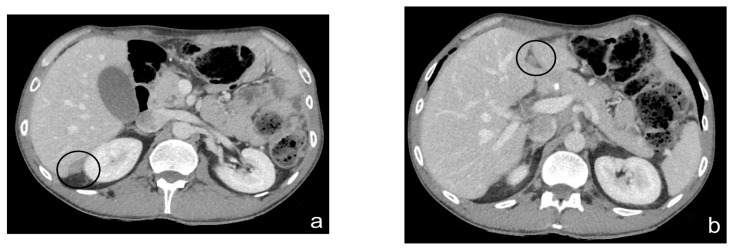

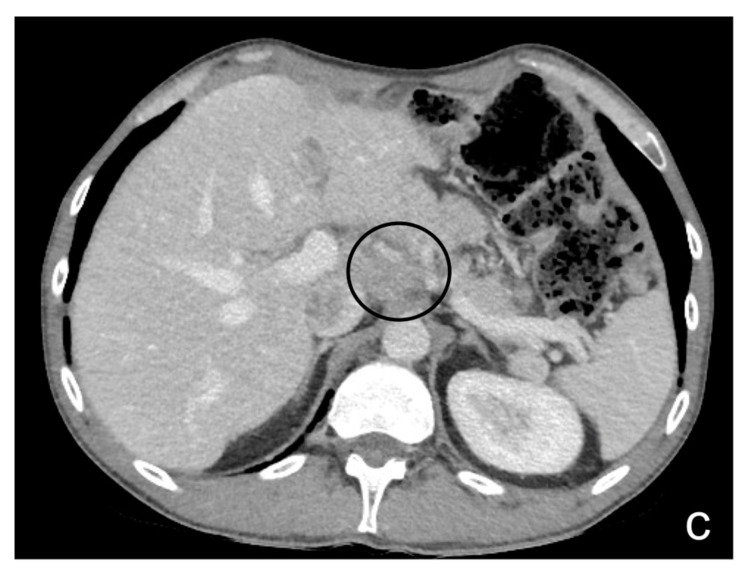
Contrast-enhanced CT scan, axial views. Case of an infiltrating-type gastric adenocarcinoma in a male, 40 y. Nodule of peritoneal carcinosis (black circle), localized on the Glissonian surface (**a**) in the fold of the falciform ligament (**b**) in the perihilar area (**c**).

**Figure 3:**
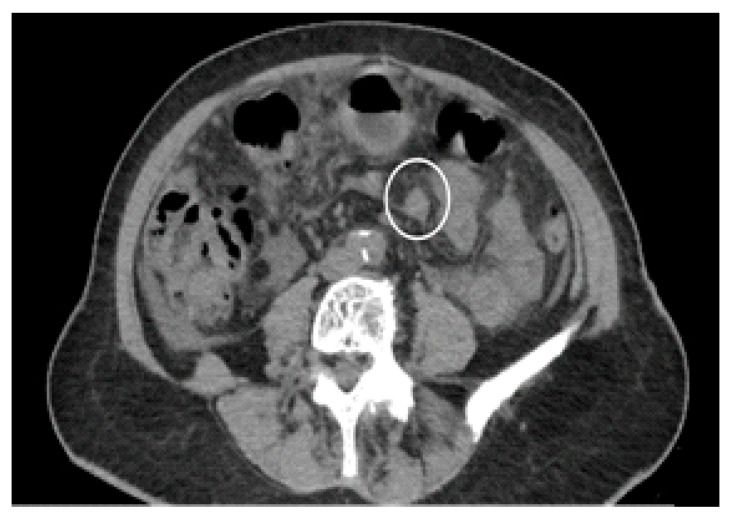
Contrast-enhanced CT scan, axial views. Case of gastric adenocarcinoma in a female, 79 y. Multiple nodules (white circle), localized in the mesogastric and ipogastric area on the anterior abdominal wall, between bowel loops and in pelvis.

The CT scan is a commonly employed imaging technique for the preoperative evaluation of patients who are selected for SRC and HIPEC [44] (Figure 2 and Figure 3).

In fact, CT has excellent spatial and temporal resolution, producing highly detailed anatomical images of the abdomen and pelvis in a few seconds. In addition, reformatted coronal and sagittal reconstructions allow multi-planar imaging without additional imaging time. However, CT scanning uses ionizing radiation [45,46].

In an analysis, Mazzei et al. [47] evaluated the accuracy of MDCT in detecting and localizing peritoneal carcinomatosis in patients with advanced ovarian cancer. The analysis was conducted at both patient and regional levels. In the patient-level analysis, CT showed sensitivity, specificity, PPV, NPV, and accuracy in the detection of peritoneal carcinomatosis of 100%, 40%, 93% 100%, and 93%, respectively. However, in the analysis at the regional level, CT showed sensitivity, specificity, PPV, NPV, and diagnostic accuracy of 72%, 80%, 66%, 84%, and 77%, respectively. These results encourage the use of MDCT, provided, however, that it is performed using an optimized dedicated protocol and analyzed by an experienced radiologist, in order to identify patients with peritoneal carcinomatosis. In fact, there is a lack of consensus regarding the acquisition protocol to be employed to illustrate peritoneal carcinomatosis during CT staging. Specifically, the appropriate role of the acquisition phases beyond the portal phase is not well defined. Certain researchers emphasized the potentially significant impact of the delayed phase in MRI scans, which enhances the sensitivity for detecting PC [48,49,50]. They concluded that it may contribute to the improved visualization of the thickened, enhanced peritoneum during the delayed phase, indicating the gradual accumulation of contrast material in the peritoneal tissues. Similar considerations have not been clearly established for CT examinations.

Although this finding might suggest the incorporation of the delayed phase into routine CT scans, it is widely acknowledged that obtaining images during an additional phase leads to increased patient exposure to ionizing radiation and prolonged examination times. Rodolfino et al., in their study [51], evaluated the diagnostic accuracy of delayed-enhanced-phase in addition to portal-enhanced-phase MDCT imaging for detecting PC implants. They found no statistically significant differences when comparing set A (portal-phase images) with set B (delayed-phase images) and set C (portal- + delayed-phase images), respectively, as assessed by experienced readers.

Furthermore, optimal small-bowel distension should, in addition, heavily improve the diagnostic accuracy of CT in the evaluation of regions 9–12. In this regard, as described by Delgado-Barriga et al. [52] patients should undergo bowel preparation two days before the examination, involving both a low-residue diet (on the first day) and a laxative formulation of sodium picosulfate with magnesium citrate (on the second day). A neutral enteric contrast agent (mannitol solution 2.5%) should be administered orally to achieve small-bowel distention. Outside the scanner room, the patient must consume 1800 mL of the oral contrast agent at a steady rate (approximately 300 mL every 10 min) over the course of 1 h. Immediately before scanning, 20 mg of an antiperistaltic agent should be injected intravenously to diminish bowel motions and related artifacts.

Recent studies also focused on the role of dual-energy CT in many fields, especially in oncology [53,54]. For example, Darras et al. [55] demonstrated that virtual monoenergetic imaging (VMI) reconstruction obtained from contrast-enhanced dual-energy CT scans of the abdomen and pelvis at 40 keV maximizes the conspicuity of metastatic peritoneal deposits and improves radiologists’ diagnostic confidence compared with conventional CT images.

However, although CT is routinely used in almost all medical centers for the evaluation of peritoneal cancer, its shortcomings in accurately detecting peritoneal tumors are well documented. The reported sensitivity of MDCT in the assessment of PC varies from 25% to 90%, depending on the location, size, and morphology of the tumor deposits and the adequacy of bowel opacification, as well as the location and possible presence of free intra-abdominal effusion [56,57].

Chua et al. [58] found that the accuracy of the identification of peritoneal lesions with CT, regardless of size, ranged from 51% to 88% in the nine abdominalpelvic regions and from 21% to 25% in the four small-bowel regions. Indeed, the suboptimal contrast resolution of CT is a major weakness in its use in the evaluation of the pelvic regions and the four critical regions of the small bowel.

The sensitivity of CT also decreases dramatically with small peritoneal tumors. Koh et al. [59] reported a CT sensitivity of only 11% for lesions < 0.5 cm in patients with colorectal cancer.

Jacquet et al. [43], in a similar analysis, reported that the sensitivity of CT in identifying lesions smaller than 0.5 cm was 28%, while for tumors measuring 0.5 cm to 5 cm it was 72%, and for tumors larger than 5 cm, the sensitivity was 90%. 

These CT limitations result in preoperative PCI that consistently underestimates the volume of peritoneal disease.

Despite the performance of CT, a previous meta-analysis on the diagnostic performances of CT and MRI (DWI) for PM detection nevertheless concluded that CT should be the preferred imaging modality for PM detection. This conclusion was drawn primarily on the basis of the robustness of the data, because the number of MRI studies included at that time was significantly lower than the number of CT studies included (3 vs. 19) [60].

However, currently, there is a growing interest in functional imaging techniques, such as PET, which may be combined with CT and magnetic-resonance imaging (MRI), especially with the addition of diffusion-weighted imaging (DWI) sequences [61].

### 2.3. Magnetic-Resonance Imaging

The use of MRI offers significant advantages in the evaluation of cancer patients and those with peritoneal metastases [62,63,64,65].

A standardized protocol, including diffusion-weighted and post-contrast injection sequences, allows the efficient exploration of small peritoneal tumors that are often not visualized by other imaging methods [66,67]. According to certain studies, pineapple juice could be used as an oral contrast agent for medical imaging. To achieve negative intraluminal contrast and expand the bowel, patients are instructed to consume one liter of pineapple juice an hour before their MRI procedure, as the juice contains high levels of manganese [68].

It is shown in the literature that peritoneal tumors show marked enhancement 5 min after gadolinium administration. This allows better identification of small tumors than when using other imaging modalities, such as CT and/or PET [69,70]. 

In a direct comparison of MRI, CT, and surgical exploration, the region-based sensitivity of MRI, enhanced with delayed postcontrast sequences, for peritoneal tumors less than 1 cm was 85–90% compared with 22–33% on CT. The average sensitivity of MRI in identifying implants of different sizes was 84% compared with 54% on CT [49].

**Figure 4:**
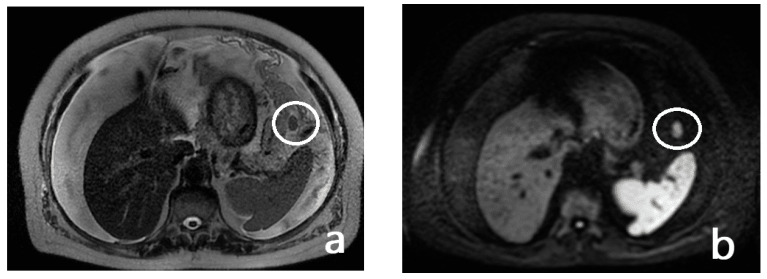
A 73-year-old man with a diagnosis of signet-cell gastric adenocarcinoma located in the greater curvature. T2w axial (**a**) and DWI b1000 axial (**b**) MR images of the upper abdomen show tumoral implants (white circles) in the gastrosplenic ligament. Note also the presence of free peritoneal fluid.

**Figure 5:**
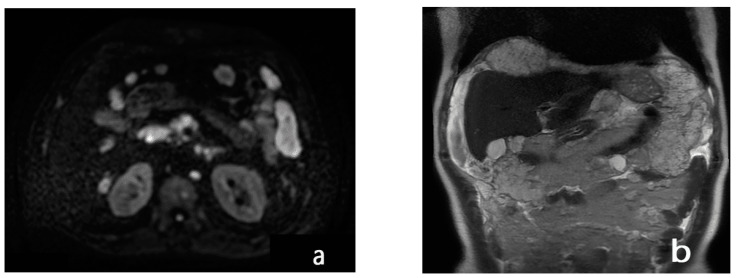
A 75-year-old man with a diagnosis of cecal adenocarcinoma. DWI b1000 axial (**a**) and T2w coronal (**b**) MR images of the upper abdomen reveal widespread nodular peritoneal carcinomatosis causing compression of the intra-abdominal organs, particularly at the level of the body of the gall bladder and cystic duct, accompanied by free fluid in perihepatic, perisplenic, and intestinal-loop locations.

In addition, the combination of morphologic sequences with diffusion sequencing demonstrated a 20% increase in sensitivity by MRI in the identification of lesions [71,72,73,74] (Figure 4 and Figure 5).

Indeed, according to Low et al. [66], on DWI, ascites and bowel contents are suppressed, while peritoneal and serosal tumors, showing diffusion restriction, are represented as areas of high signal intensity. 

However, the limitations of DWI sequences include the T2-shine-through effect typical of cystic lesions. The generation of an apparent diffusion coefficient (ADC) map, which demonstrates only true limited diffusion, can eliminate this T2 signal [75]. Radiologists, therefore, should become familiar with normal structures that show limited diffusion, including benign lymph nodes, intestinal mucosa, and spleens. Limited diffusion is not specific for malignancy and can also be seen in inflammation and ischemia [76]. 

A prospective study [77] demonstrated the diagnostic value of MRI to be superior to that of CT and PET-CT in assessing peritoneal staging in patients with ovarian cancer, reporting accuracies of 91%, 75%, and 71%, respectively. In particular, mesenteric and serosal deposits, as well as subcentimetric lesions, were better described by MRI with DWI functional sequences than by CT.

All images should, however, be acquired with restrained breathing to minimize respiratory artifacts, which may obscure thin peritoneal tumors. In addition, the preparation of the patient with bowel contrast is an essential element, as is the use of pharmacologic agents to decrease peristalsis. Indeed, a collapsed bowel creates problems for image interpretation, as it can mask thin peritoneal tumors or inflammation involving the intestinal serosa, mesentery, or adjacent peritoneum. In addition, an undistended small bowel may be interpreted as an abdominal mass, thus generating false positives. Adequate bowel distension is, therefore, an essential element in the peritoneal-MR-imaging protocol as it improves accuracy and confidence in image interpretation [77]. 

Additionally, MRI can detect recurrences, sometimes even before serial tumor markers [78].

Finally, new imaging techniques have been developed in the field of oncology [79,80]. Among these, dynamic contrast-enhanced (DCE) MRI has proven useful in some studies in determining the extent of PC in correlation with surgical and histopathological findings [81].

Therefore, overall, MRI has many advantages, but also limitations. Some of these limitations, such as its limited availability, high cost, and longer examination times are well known. The MRI radiologist requires additional time, as well as considerable training, to learn the subtleties of peritoneal MRI interpretation [82,83]. 

Therefore, although, in clinical practice, CT is performed as a first-level investigation and MRI as a second-level examination in order to increase diagnostic power when CT results are equivocal, the two methods can be considered complementary. In fact, MRI has been shown to be useful in patients with an intermediate burden of peritoneal metastases in order to identify individuals with resectable disease, as well as in identifying small peritoneal nodular lesions < 1 cm in size and in detecting small-bowel involvement (9–12 Sugarbaker regions); the latter still remains a challenge for the radiologist, and it is one of the main causes of incomplete cytoreduction.

### 2.4. PET-CT

**Figure 6:**
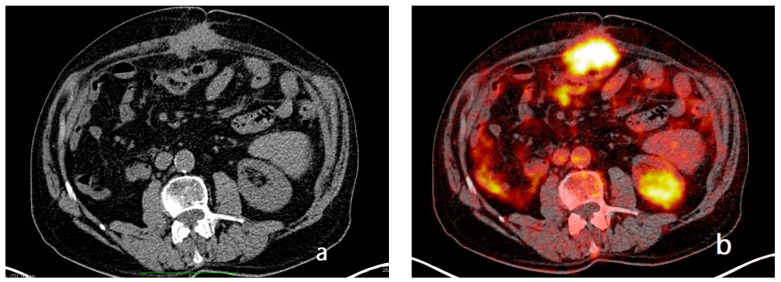
Axial non-contrast-enhanced computed tomography through the mid-abdomen demonstrates a nodule of peritoneal carcinosis on the middle abdominal wall (**a**) in a 68-year-old man. 18F-fluorodeoxyglucose PET with low-dose attenuation-correction-computed-tomography-fused image demonstrates its fluorodeoxyglucose uptake (**b**).

Functional imaging techniques, such as PET-CT and DWI-MRI, seem to overcome some of the limitations of CT. Both techniques, in fact, show high contrast resolution between tumors and normal tissues [84]. In particular, PET-CT findings report not only detailed anatomical data, but also data on increased glucose metabolism in tumors [Figure 6]. In addition, because PET-CT is a full-body-imaging technique, it is capable of detecting distant metastases. Its main disadvantages, however, are its availability, higher cost, and the limited imaging it offers of small tumor volumes (the current spatial resolution is 4 mm). 

The comparison of imaging modalities can be challenging. The performance of each modality may depend on the protocol used, the type of tumor (mucinous/nonmucinous and invasiveness), and the radiologist’s level of experience. In studies comparing imaging outcomes with surgical outcomes, there was no benefit of PET-CT over CT alone in terms of patient management [85,86].

In their study, Elekonawo et al. investigated the possibility of assessing the extent of peritoneal disease in patients with rectal cancer by PET-CT. The results demonstrated that PET-CT underestimates the extent of PC compared with intraoperative findings, which was in line with the results reported in the literature [87,88]. 

Instead, Dromain et al. concluded that neither CT nor PET-CT examination was a reliable imaging method in the preoperative assessment of the extent of peritoneal involvement in colorectal cancer, particularly when assessing small-bowel involvement [82]. 

The limitations of PET-CT include false-negative interpretations due to small lesions that do not show FDG uptake, or that may be obscured by normal bladder or bowel activity. The lower cellularity of mucinous metastases is another source of false negatives in interpretations with PET [89].

The false-positive interpretations using PET decrease its specificity, with potential over-staging of disease. False-positive PET findings may reflect an inflammatory reaction of the peritoneum adjacent to large or multiple tumor implants, the presence of foreign bodies, or inflammatory reactions due to a previous surgery or normal physiological activity in the bowel. However, PET-CT remains the best imaging modality for depicting extraperitoneal metastases [90].

## 3. What the Radiologist Should Know about Radiologic Findings

### 3.1. What Are the Main CT and MRI Findings on Which the Radiologist Should Focus?

#### 3.1.1. Establish the Presence of Peritoneal Metastases

Each radiologist must first assess the presence or absence of peritoneal implants, either by CT or MRI, after which he or she must evaluate their location and characteristics (number, morphology, volume and density or intensity before and after the intravenous injection of contrast agents).

The most frequent findings, in the case of PC, vary from multifocal nodules to masses infiltrating the peritoneal cavity, omental thickening, ascites, peritoneal nodular thickening, and peritoneal enhancement [91].

Peritoneal implants may be solid, cystic, partly solid and partly cystic (mixed), or calcific; they may present as nodules with well-defined margins, round or oval in shape, or with indefinite, spiculated contours [92]. 

In addition, the radiologist should provide at least a semi-quantitative assessment, describing the peritoneal tumor as diffuse, multifocal, or localized, and report the total number of regions involved, up to 13 [93]. 

**Figure 7:**
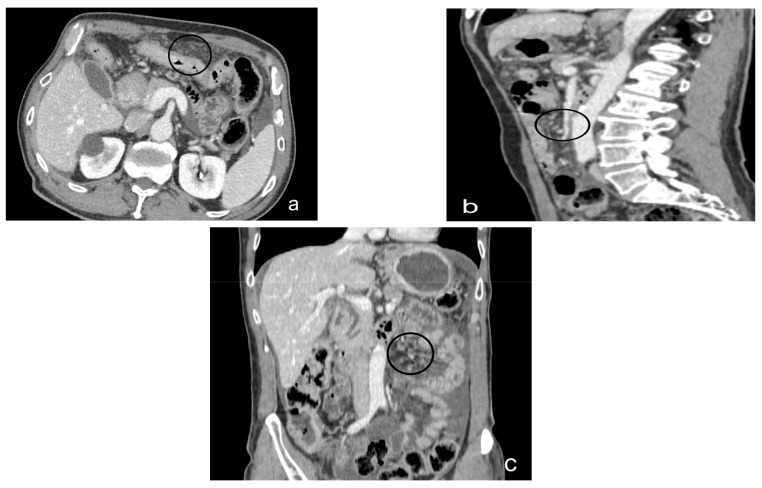
Contrast-enhanced CT scan, axial view (**a**), sagittal view (**b**), and coronal view (**c**). Case of micronodular peritoneal carcinomatosis (black circle), in a male, 75 y, affected by cardias’ adenocarcinoma T4. Diffuse peritoneal involvement with micronodular lesions and ascitis.

**Figure 8:**
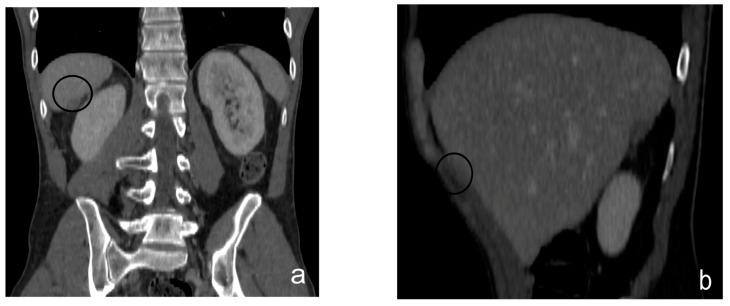
Contrast-enhanced CT scan, coronal view (**a**) and sagittal view (**b**). Nodule of peritoneal carcinosis (black circle), localized on the Glissonian surface.

**Figure 9:**
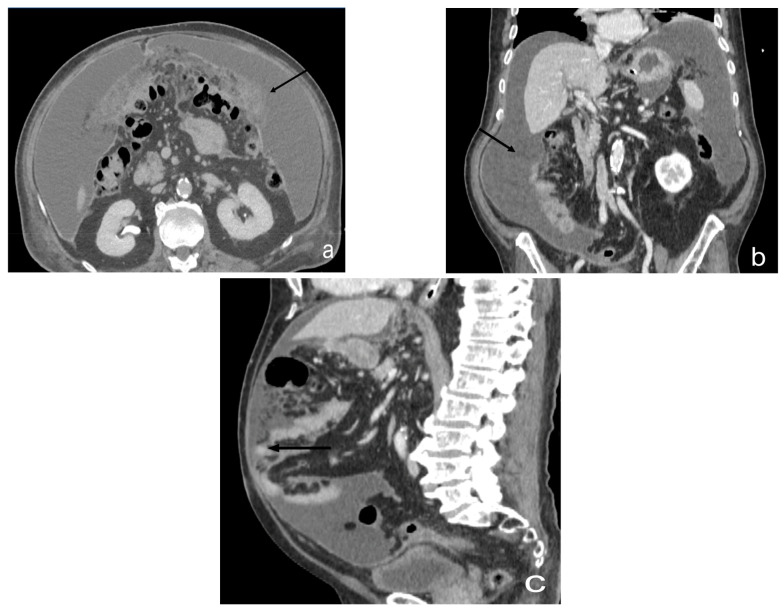
Contrast-enhanced CT scan, axial view (**a**), coronal view (**b**), and sagittal view (**c**). Case of ascitis and omental cake in a male, 76 y. Massive ascitic effusion (black arrow) in the supramesocolic and submesocolic region, with thickening of the omentum and nodular appearance of “omental cake.”

**Figure 10:**
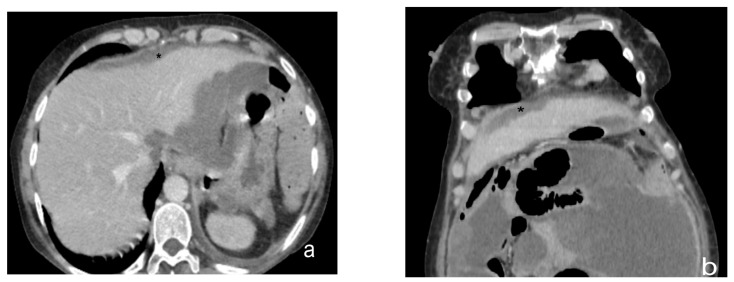
Contrast CT scan, axial view (**a**) and coronal view (**b**). Case of a plaque-like pattern in peritoneal carcinomatosis. Peritoneal thickening (black star) located on the lower surface of the right diaphragm, depressing the liver surface.

**Figure 11:**
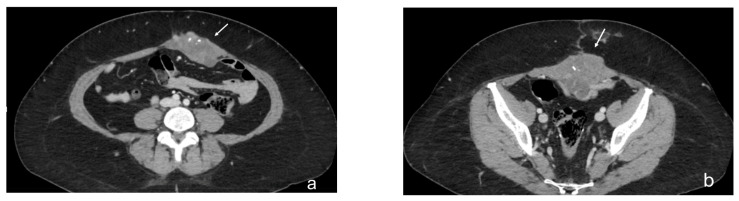
Contrast-enhanced CT scan, axial views (**a**,**b**). Case of a mass-like pattern of peritoneal carcinomatosis. Nodule (white arrow) located in the left rectus muscle of the abdomen, as a result of the confluence of multiple nodular lesions.

**Figure 12:**
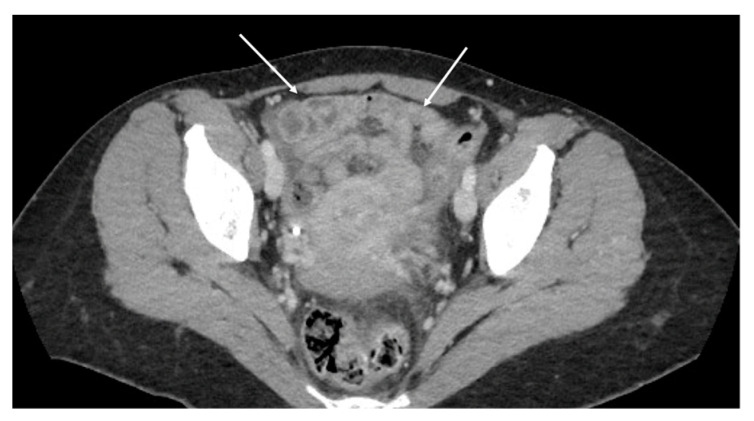
Contrast-enhanced CT scan, axial view. Case of theca pattern. Nodular thickening of the peritoneum lining the loops of the small bowel in pelvis (white arrows).

A further task of the radiologist is to describe the distribution pattern. Several patterns have been identified in the literature:

- Micro-nodular pattern: characterized by the visibility of micro-nodules with a diameter between 1 and 5 mm, which may diffusely involve the mesentery, small and large omentum, serous tonaca, and subserous fat [93] (Figure 7).
- Nodular pattern: characterized by the presence of nodular implants >5 mm in diameter (Figure 8).
- “Omental cake”: in radiology, “omental cake” describes the nodular thickening of the omentum, leading to the posterior dislocation of the bowel relative to the anterior abdominal wall [94] (Figure 9).
- Plaque pattern: confluent nodular tumor implants that are typically located on the lower surface of the right diaphragm and may manifest as a depression of the liver surface, mimicking capsular or subcapsular liver metastases. They present as areas of low attenuation relative to the parenchyma on postcontrast scans [94,95] (Figure 10).
- Mass-like pattern: the mass-like pattern results from the confluence of multiple nodular implants and can lead to the formation of a mass of tissue that can reach sizes of several centimeters. When the diameter of a mass reaches 10 cm, it is referred to as a “bulky tumor” [47] (Figure 11).
- Theca pattern: characterized by a nodular thickening of the visceral peritoneum lining the loops of the small bowel. Sometimes, this heteroplasic thickening generates narrowing, with the consequent obstruction and dilation of the proximal loops, a condition also referred to as “frozen pelvis” [95] (Figure 12).

The presence of free peritoneal fluid or ascites is another frequent finding in patients with peritoneal carcinomatosis. This may be related to increased capillary permeability and/or fluid production, as well as to lymphatic vessel obstruction or decreased absorption. Localized ascites is also a sign suggestive of carcinomatosis [96]. 

**Figure 13:**
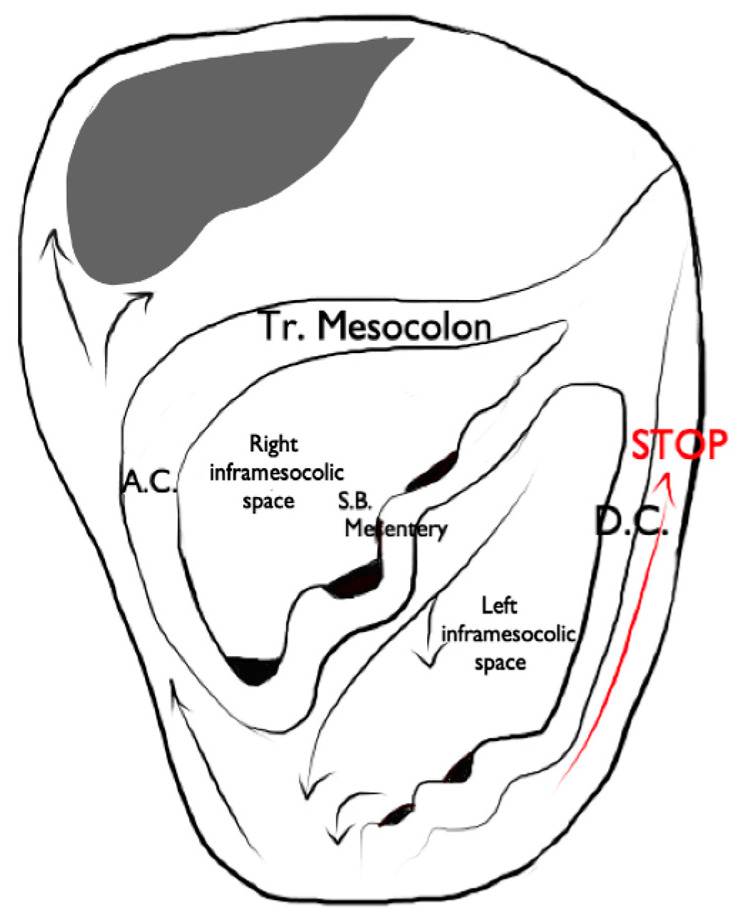
Illustrated representation of peritoneal fluid circulation, indicated by black arrows. It is important to note how the left phrenicocolic ligament (red arrow) represents a stopping point for the circulation of peritoneal fluid and, consequently, for metastatic diffusion. A.C. (ascending colon), Tr. Mesocolon (transverse mesocolon), D.C. (descending colon), S.B. Mesentery (small-bowel mesentery).

The distribution of peritoneal carcinosis is clearly related to ascitic fluid dynamics. Four predominant sites of peritoneal metastasis are identified: the recto-vescical (Douglas) excavation, the right lower quadrant, at the lower end of the right infra-colic space along the root of the small0bowel mesentery, the sigmoid mesocolon, and the right para-colic shower [97] (Figure 13). 

The locations of peritoneal implants are extremely important for the pre-surgical and pre-treatment evaluation of PC. Radiologists must specify each site of peritoneal carcinomatosis in order to provide the most accurate staging possible. To obtain an accurate assessment of PC and to map peritoneal implants, radiologists should commonly use the Sugarbaker-proposed assessment system, which allows calculation of the Peritoneal Cancer Index (PCI) [98,99].

This index divides the entire abdominal and intestinal region into 13 regions. In each of the 13 regions, the radiologist should report the maximum size of the measured visible lesion and assign a score based on the largest lesion size. This score ranges from LS = 0 to LS = 3. The LS = 0 means no visible tumor, LS = 1 means a tumor-lesion size of less than 0.5 cm, LS = 2 means a tumor-lesion size between 0.5 cm and 5 cm, and LS = 3 means a tumor-lesion size greater than 5 cm, or confluent lesions [22]. 

The accurate depiction of the sizes and locations of peritoneal metastases in the 13 abdominal and pelvic regions may allow the radiologist to calculate a radiological PCI. The latter requires an accurate assessment of the entire peritoneal cavity, including the parietal peritoneal surfaces and the visceral peritoneum, including the intestinal serosa and mesentery. Finally, the determination of tumor resectability based on these preoperative imaging findings may prevent unnecessary laparotomy in patients whose excessive tumor burden or involvement of critical structures would preclude successful cytoreduction [46].

#### 3.1.2. What a Radiologist Needs to Report: “Structured Reporting”

Structured reporting in radiology is a uniform and comprehensive approach to the interpretation of medical imaging, ensuring that standardized reporting methods are employed. 

Structured radiological reports on peritoneal carcinosis commonly encompass a comprehensive analysis of the peritoneal cavity, including tumor identification and localization, the evaluation of the extent of the disease, and the assessment of treatment response [100,101].

For lesions, it is important to include the number, the morphology, the volume, and the density/intensity. The findings can include multifocal nodules, infiltrating peritoneal cavity masses, omental nebulosity, ascitis or free peritoneal fluid, nodular peritoneal thickening, and peritoneal enhancement.

The patterns included in these reports are micro-nodular, nodular, mental cake, plaque pattern, mass-like pattern, and theca pattern. It is also important to include the contraindications of cytoreduction, such as the involvement of the root of the mesentery, the involvement of the hepatic hilum and hepatoduodenal ligament, and full diffuse pelvis infiltration. The radiologist should also include the Peritoneal Cancer Index in the report (Table 1).

The implementation of structured reporting for peritoneal carcinosis can enhance communication between radiologists and referring physicians, fostering improved consistency and accuracy in the diagnosis and management of the disease. Additionally, structured reporting for peritoneal carcinosis contributes to the minimization of the variability in interpretations and enhances the overall quality and efficiency of patient care [102].

## 4. What the Radiologist Needs to Know about Treatment

Peritoneal tumors have traditionally been associated with significant morbidity and mortality; however, the management of these tumors has evolved substantially. Advanced treatment options, including cytoreductive surgery and intraperitoneal chemotherapy, have significantly improved long-term patient survival, while radiotherapy, unlike other pathological conditions, is not generally used as a treatment [98]. The role of the radiologist is critical in selecting patients with resectable disease for whom complete cytoreduction can be achieved.

The rationale for HIPEC is based on the direct cytotoxicity of hyperthermia against malignant cells, combined with the cytotoxic effects and pharmacokinetic advantages of the intraperitoneal route of heat-enhanced chemotherapeutics. The aim of locoregional treatments is to achieve a high and persistent tumor drug concentration while limiting systemic concentration. It is critical to ensure that the residual tumor deposition after CRS is <2.5 mm in order to allow subsequent HIPEC to be optimal, as even the most ambitious perfusion strategies penetrate only a few millimeters [103]. 

The application of HIPEC involves the instillation of a heated chemotherapeutic agent (at a temperature of 41–43 °C) directly into the abdominopelvic cavity immediately after the completion of surgery. This procedure delivers a high dose directly to the site of any residual microscopic tumor cells while minimizing systemic toxicity [104,105].

Therefore, IP chemotherapy is an ideal method because it offers a number of advantages: (1) intraperitoneal infusion drugs work immediately on both metastatic lesions on the peritoneal surface and on free tumor cells in the peritoneal cavity; (2) compared with intravenous chemotherapy, IP chemotherapy generates a higher concentration of drug in the abdominal cavity; and (3) some agents are not readily absorbed into the systemic circulation, causing a prolonged half-life in the abdominal cavity and less systemic toxicity [106].

To ensure that patients benefit from this aggressive multimodal treatment, it is imperative to select individuals with resectable peritoneal disease for whom complete cytoreduction can be achieved. Therefore, four main areas for the assessment of patients’ suitability to undergo cytoreductive surgery have been described: clinical evaluation, histopathologic evaluation, radiologic evaluation, and PCI [107].

Many studies have demonstrated the effectiveness of combining cytoreductive surgery with HIPEC in carefully selected patients to improve overall survival. However, it is challenging to generalize these findings to the general population due to the fact that each study focused on a different primary tumor site. For instance, in [108] 298 patients from 16 different centers were analyzed, and it was observed that the integrated approach could be executed safely with satisfactory morbidity and mortality rates in a specialized unit environment, with 63% of the patients achieving survival beyond 10 years. The authors’ conclusion suggests that reducing non-definitive operative and systemic chemotherapy interventions prior to definitive cytoreduction may improve the practicality and enhance the results of this treatment in order to attain long-term survival. They observed that achieving optimal cytoreduction yields the most favorable outcomes.

Furthermore, other treatments have been studied and, in the literature, we found many examples. One of these treatments is described in the study by Coccolini et al. [109], who present a new drug for the intraperitoneal treatment for GC, developed in Germany, whose name is catumaxomab (marketed as Removab^®^), a chimeric monoclonal antibody derived from a combination of rat and mouse antibodies. Specifically, catumaxomab is employed in the treatment of malignant ascites. In a phase III randomized trial, the intraperitoneal administration of this anti-EpCAM antibody demonstrated significant benefits in terms of puncture-free survival (survival without repeated paracentesis) for patients with malignant ascites [110]. While no statistically significant increases in median overall survival were observed for other cancers, a slight survival improvement was associated with the use of catumaxomab in patients with gastric cancer (GC) [110]. 

This is an important and challenging field of study, and it is imperative that researchers continue to search for the treatments that offer the greatest benefits to patients. 

### Qualitative Analysis of Lesions and Non-Resectability Criteria

The radiologist should initially rule out extra-abdominal metastatic disease, including the pleural extension of the disease, and carefully evaluate the liver for metastatic disease (which is a contraindication, with the exception of colorectal liver metastases). The radiologist should then carefully evaluate the lesions and describe their relationships and/or possible involvement with neighboring vascular structures (aorta, vena cava, other vessels) [107,111]. 

**Figure 14:**
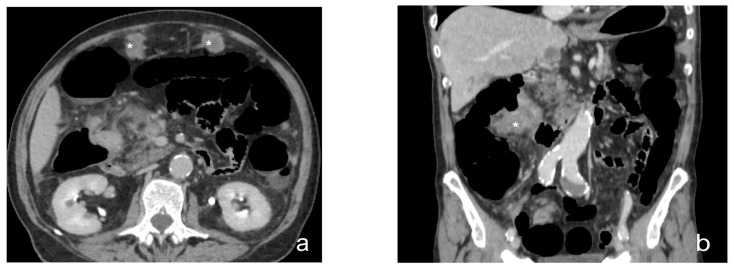
Contrast-enhanced CT scan, axial view (**a**), coronal view (**b**). Case of involvement of the root of the mesentery in a male, 63 y. Multiple and widespread peritoneal nodules (white star) present in all abdominal quadrants, with the largest in the mesogastric area.

**Figure 15:**
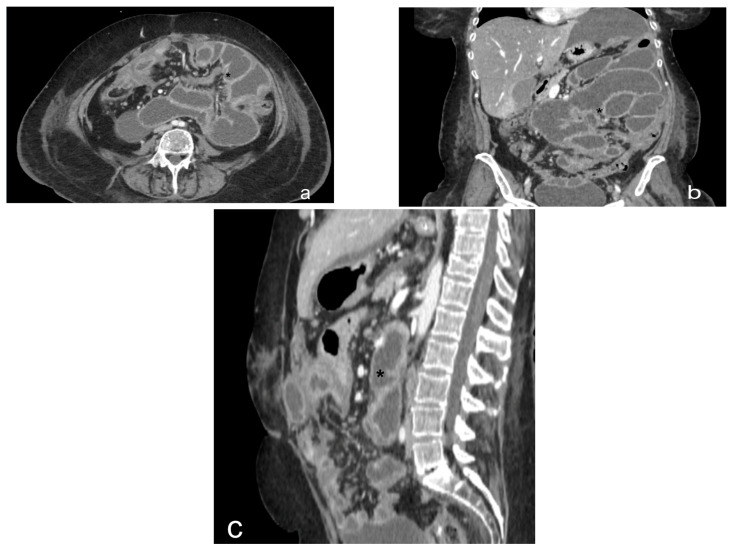
Contrast-enhanced CT scan, axial view (**a**), coronal view (**b**), sagittal view (**c**). Case of ovarian carcinoma in a female, 50 y. Peritoneal carcinosis nodules (black star) involving fascia trasversalis and the ileal loops in the right flank, causing fluid distension of the proximal loops.

The radiologist should also evaluate additional findings that represent a contraindication to complete cytoreduction, which are:

- Mesenteral root involvement
  Disease in the small bowel and intestinal mesentery constitutes a sentinel and limiting criterion in decision making in CRS. Therefore, the evaluation of small-bowel loops and their mesentery should be a key component in the preoperative imaging evaluation of a patient with PC [24]. The diffuse involvement of the mesentery root is, in fact, a criterion for unresectability. Thin mesenteral tumor sheets are invisible on CT and PET scans [111]. In a study of 30 cases by Dromain et al. [87], CT detected implants in the small bowel in 26% of cases, whereas the true incidence of this disease location at the time of surgery was 83%.
  Furthermore, MRI has been reported to be superior to CT in the evaluation of the intestinal tract and mesenteric involvement in light of its high capacity in soft-tissue studies [112].
  Diffuse small-bowel involvement (mesentery and/or intestinal serosa) remains difficult to represent, however, and when present, the radiologist should estimate the extent of involvement as less than or greater than 50%. The radiologist should also describe the number and location of any stenosis of segments of the small intestine, as well as invasion of the colon or gastric system, to produce a complete analysis of the entire digestive tract [69].
  Indeed, Jacquet et al. [43] found that when preoperative CT showed a tumor causing intestinal obstruction, surgical cytoreduction was suboptimal in 88% of cases. If the tumor obstructing the jejunum or superior ileum was greater than 5 cm in diameter, no subject had a complete CRS.
  In fact, the two radiologic findings that Sugarbaker et al. [113] reported as most strongly associated with poor outcomes in CRS are the presence of tumor nodules greater than 5 cm on small-bowel surfaces and segmental small-bowel obstruction.
  Preoperative imaging can aid in patient selection by avoiding surgery for patients whose tumors are excessively large for adequate surgical cytoreduction; in particular MRI using gadolinium and DWI sequences routinely describe tumor-cell sheets involving the serosa of the small intestine and mesentery, which are typically not seen on CT or PET [55] (Figure 14 and Figure 15).

- Hepatic port and hepato-duodenal ligament
  The involvement of the hepato-duodenal ligament or hepatic hilum is a further criterion of unresectability. The radiologist’s task is to evaluate direct signs of tumor infiltration, such as the disappearance of a clear peri-portal fat plane due to tumor replacement and/or indirect signs of biliary and vascular stenosis [66].

- Diffuse infiltrated pelvis (frozen pelvis)
  A diffusely infiltrated pelvis (known as frozen pelvis) is a condition that precludes cyto-reductive surgery, as well as suggesting the extensive involvement of the bladder and trigonal region. The involvement of the pelvis is best studied with MRI, which is the most sensitive imaging method, thanks to its high-resolution soft-tissue contrast and multi-planar capabilities. In fact, in the absence of ureteral dilatation, this tumor is very difficult to represent on CT or PET-CT [114,115].

Radiology reports are critical in patient management because they allow the determination of the most appropriate form of patient management. Traditional reports are associated with excessive variability in language, length, and style, which can reduce their clarity and make it difficult for referring physicians to identify key information for patient care. Structured reporting has been recommended as a potential solution to improve the quality of radiology reports [116,117]. Even in the case of CP, we believe that it is necessary to have a complete, accurate, and universal radiological lexicon, in order to standardize the language and make radiological reports more synthetic and clear. 

## 5. What Radiologists Need to Know about Key Differential Diagnoses

### 5.1. Peritoneal Malignant Mesothelioma

Malignant mesothelioma is an uncommon malignant neoplasm that arises from mesothelial cells or multipotent subcutaneous mesenchymal cells of the pleura, peritoneum, or pericardium, or the tunica vaginalis of the testis. Most malignant mesotheliomas originate in the pleura [117,118].

Primary peritoneal mesotheliomas account for 6–10% of malignant mesotheliomas [119]. 

The association between malignant mesothelioma and asbestos exposure is well known [120]. 

Pleural plaques are present in approximately 50% of patients with malignant peritoneal mesothelioma [121].

Malignant peritoneal mesothelioma presents with two main patterns: the focal form and the diffuse form. The focal form manifests as a large mass, usually located in the upper abdomen, with additional scattered peritoneal nodules. The diffuse or desmoplastic form, on the other hand, manifests as a diffuse peritoneal thickening without a well-defined mass; this form tends to spread along the serosal surfaces and encompasses both solid and hollow visceral organs [122]. 

Omental cake and ascites are usually present [117]. 

The stellate infiltration of the mesentery is common, and appears as increased mesenteric fat attenuation, perivascular soft-tissue thickening, and vascular-bundle stiffness [123].

The presence of peritoneal masses with or without ascites in the abdominal cavity is more suggestive of peritoneal carcinomatosis in most patients. Malignant mesothelioma should be considered when the predominant finding on imaging is sheet-like thickening of the peritoneum, and when there is a history and imaging finding of asbestos exposure. Malignant peritoneal mesothelioma is, however, indistinguishable from carcinosis when multifocal peritoneal nodules and/or an omental-cake picture are visible on imaging. To support the diagnosis of malignant mesothelioma, a lack of evidence of primary malignancy or extraperitoneal metastases and the absence of lymphadenopathy are crucial. Calcifications are further, rarely encountered findings in MM.

### 5.2. Primary Peritoneal Serous Carcinoma

Primary papillary serous carcinoma of the peritoneum is a rare neoplasm that predominantly affects postmenopausal women [124].

Widespread peritoneal involvement is typical, particularly that of the omentum. Extensive calcification of the omental envelope is present in many cases and is a useful CT finding to rule out mesothelioma. In addition, the absence of an ovarian mass is critical in ruling out papillary serous ovarian carcinoma, which otherwise looks similar on CT and is histologically identical to its primary peritoneal counterpart.

### 5.3. Desmoplastic Small Round-Cell Tumor

Desmoplastic small round-cell tumors (DSRCTs) are rare but extremely aggressive neoplasms that occur primarily in adolescents and young adults [125].

The CT features of DSRCT include multiple intra-abdominal soft-tissue masses involving the omentum and serosal surfaces without an apparent organ of origin. Punctate calcification or necrosis in the mass, ascites, liver metastasis, lymphadenopathy, bowel obstruction, and hydronephrosis are also seen in patients with DSRCT [126,127].

### 5.4. Disseminated Peritoneal Leiomyomatosis

Disseminated peritoneal leiomyomatosis (DPL) is usually discovered incidentally during surgery or imaging examinations in women of childbearing age with a history of uterine leiomyomas. It may be associated with the elevated estrogenic conditions caused by pregnancy or oral contraceptive use [128]. 

On CT examination, DPL is characterized by solid masses of muscle-like density, well circumscribed with smooth contours and delayed enhancement, without evidence of omental nebulosity or ascites [129].

Imaging features, a close or remote history of cesarean delivery or hysterectomy, and the presence of a uterine leiomyoma are the clues that direct us to the diagnosis of LPD. Furthermore, peritoneal leiomyomas regress spontaneously or after the discontinuation of ovarian hormones in most patients.

In rare cases, however, sarcomatous degeneration may occur [130].

### 5.5. Pseudomyxoma Peritonei

**Figure 16:**
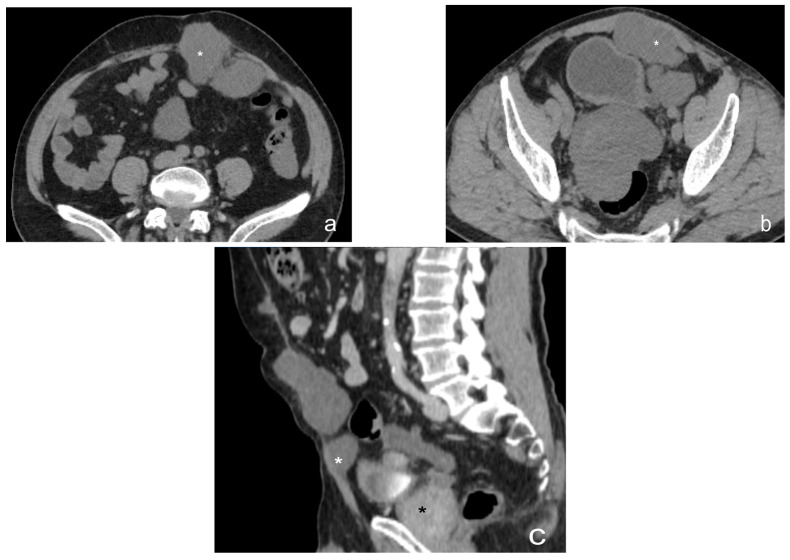
Contrast-enhanced CT scan, axial view (**a**,**b**) and sagittal view (**c**). Case of pseudomyxoma peritonei in a male, 68 y with left-colon adenocarcinoma, IV stage. Multiple peritoneal nodules (white star) involving the anterior abdominal wall (**a**–**c**) and a major nodule (black star) localized in pelvis, compressing the sigma.

**Figure 17:**
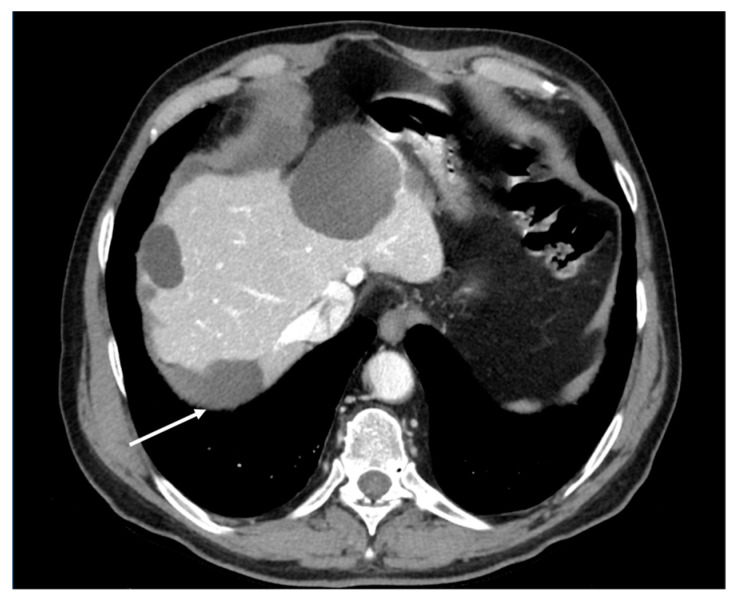
Contrast-enhanced CT scan, axial view. Case of pseudomyxoma peritonei, in a female 63 y. Lesions (white arrow) on the hepatic glissonian surface.

Pseudomyxoma peritonei (PP) is a clinical or radiologic term rather than a pathologic diagnosis, and refers to a condition in which a large amount of mucinous material spreads into the peritoneal cavity (Figure 16 and Figure 17).

Furthermore, PP frequently results from appendicular mucinous neoplasms in men and ovarian mucinous tumors in women, and the fluid may or may not contain malignant epithelial cells, depending on the primary lesion [131]. 

On ultrasonography, pseudomyxoma peritonei might be suspected when the ascitic fluid is echogenic, a finding that suggests that the fluid is gelatinous/corpusculated. In contrast to echoes that may be secondary to protein exudates, blood, or infection, pseudomyxoma peritonei echoes are not mobile [9,36]. Echogenic septates within gelatinous ascites are frequently observed. The bowel is often displaced posteriorly and assumes a stellate appearance [132]. 

Scalloping of the visceral surfaces of intraperitoneal organs is another important diagnostic factor that helps to differentiate pseudomyxoma from simple ascites. Scalloping refers to indentations occurring on the capsular margins of the intraperitoneal organs, and it is characterized by extrinsic pressure exerted by intraperitoneal mucinous implants. It is most commonly observed along the margins of the liver and spleen [133]. 

Pseudomyxoma peritonei typically does not invade visceral organs or spread via lymphatics or hematogenous routes [134].

This differentiates it from mucinous carcinosis, which tends instead to involve the thorax more frequently with effusions or pleural masses, and it may also be accompanied by mesenteric or retroperitoneal lymphadenopathy, omental cake, and parenchymal organ invasion [135]. 

The pleural extension of peritoneal pseudomyxoma is rare and may be the consequence of cytoreductive surgery and subphrenic peritonectomy, or it may be secondary to congenital pleuroperitoneal communication [136].

### 5.6. Lymphomatosiss

Although primary lymphomas can develop on the peritoneal surface as a primary process, almost all PLs are secondary to a preexisting lymphoma [131]. 

The CT findings frequently observed in PL are similar to those in PC: a diffusely thickened peritoneum, ascites, omental nebulosity, and multifocal nodules or masses in the peritoneal cavity [137,138].

However, the presence of coexisting splenomegaly and extensive lymphadenopathy are imaging features that suggest PL rather than PC.

### 5.7. Tuberculosis

Mycobacterium tuberculosis can reach the peritoneal cavity through the direct invasion of the peritoneum from the intestine or through lymphatic spread [139]. 

The CT findings of PT, such as masses and/or nodules of solid tissue in the peritoneal cavity, retroperitoneal lymphadenopathy with low attenuation, ascites, and infiltration into the omentum, may mimic PC [131]. 

However, the low-density center, due to caseous necrosis, in peritoneal masses is considered one of the typical findings of abdominal tuberculosis [140].

In addition, hepatic or splenic micro-abscesses, splenic or lymph-node calcifications associated with splenomegaly, are CT features that suggest TP rather than PC [141].

## 6. What Radiologists Need to Know about Radiomics and PCI

Radiomics, an emerging field involving the conversion of digital medical images into extractable data, data analysis, and improved medical decision making, has attracted increasing attention in recent years [142,143,144,145,146]. With radiomics, the accuracy of the diagnosis, prognosis, and prediction of treatment response can be improved, especially in the field of oncology [147,148]. Radiomics enables the noninvasive profiling of tumor heterogeneity through the extraction of complex imaging features [149,150,151]. It also enables the evaluation of changes induced in tumors by therapy (delta radiomics) [23,152]. The applications of radiomics focus mainly on diagnosis and, thus, the detection of tumors, lymph-node and/or distant metastases, and the classification of tumor histotypes, but also on survival and the assessment of responses to treatment [153].

In addition, with such techniques, it is possible to identify the correlation between the data extracted from images and the molecular and genomic characteristics of tumors (radiogenomics) for the purpose of extracting information on disease aggressiveness, prognosis, and therapeutic response [154,155].

Few studies have focused on the management of patients with peritoneal carcinosis using radiomics-based clinical-practice-decision-support tools [156,157,158].

Liu et al. used radiomic models for the preoperative prediction of occult peritoneal metastasis (PM) in advanced gastric cancer (AGC) using CT images. They were able to show that the radiomic analysis of venous CT based on the primary tumor provided valuable information with which to predict occult PM in AGCs [158].

Similarly, Song et al. developed a multi-sequence magnetic-resonance imaging (MRI)-based radiomic signature to preoperatively predict peritoneal metastasis (PM) in ovarian cancer (OC) [159].

In this regard, further discussions should be devoted to the limitations and challenges posed by the use of radiomics and artificial intelligence. In particular, the majority of the studies conducted so far have been retrospective in nature, rather than prospective; there is a need to continue demonstrating the effectiveness of AI through peer-reviewed studies, rather than relying on non-peer-reviewed journal publications. Additionally, it is important to consider that accuracy in research does not always reflect clinical efficacy, especially regarding the implementation of radiomics and AI in clinical practice. This is due to logistical difficulties and the requirement to ensure that the use of algorithms can genuinely benefit patients [160,161].

In the era of precision medicine, the creation of models to support clinical practice will hopefully improve diagnostic accuracy and prognostic power. However, further studies are needed for radiomics to enter daily clinical practice.

## 7. Conclusions

In the light of the information and considerations in this study, the centrality of imaging in the diagnosis and quantification of lesions in peritoneal carcinosis and the role of the radiologist in the multidisciplinary management of patients with this condition emerged. To this end, it is necessary for the radiologist to possess correct knowledge of the pathophysiology of this condition, of the main underlying neoplasms, of the typical imaging findings, and of the differential diagnoses, as well as of the advantages and disadvantages of the individual methods. Finally, the advent of artificial intelligence in oncology has completely revolutionized the clinical management of these patients [162,163,164,165,166,167,168,169,170]. The interconnection between structured reporting and artificial intelligence is indeed redefining the future of precision medicine, the purpose of which is to personalize treatment, as well as to improve diagnostic accuracy and prognostic power [171,172,173,174].

## Figures and Tables

**Table 1:** Peritoneal carcinosis structured report, proposed by SIRM Foundation Program.

Field	Detail	Admitted Values
Clinical	symptoms	nausea
		vomiting
		abdominal pain
		other
	laboratory	CEA
		CA 19.9
		other
Lesions	Number simptoms	numeric value
	Morphology	round
		oval
		other
	Volume	numeric value
	Density o intensity	numeric value
Findings	Multifocal nodules	yes/no
	Infiltrating peritoneal cavity masses	yes/no
	Fat stranding	yes/no
	Ascitis or free peritoneal fluid liquido peritoneale libero	yes/no
	Nodular peritoneal thickening	yes/no
	Peritoneal Enhancement	yes/no
Patterns	Micronodular	yes/no
	Nodular	yes/no
	Omental cake	yes/no
	Plaque pattern	yes/no
	Mass like pattern	yes/no
	Theca pattern	yes/no
Areas most affected	Douglas rectum-bladder excavation yes/no	yes/no
	Right lower quadrant	yes/no
	Lower part of the right infracolic space around the root of the mesentery of the small intestine, the sigmoid colon, right paracolic groove	yes/no
Contraindications to cytoreduction	Involvement of the root of the mesentery	yes/no
	Involvement of the hepatic hilum and hepatoduodenal ligament	yes/no
	Pelvis diffusely infiltrated	yes/no
PCI	The Peritoneal Cancer Index (PCI) is a quantitative scoring system that incorporates both the size and distribution of carcinoma implantation in 13 specific regions within the abdomen and pelvis. These regions, numbered 0 to 8, divide the abdomen and pelvis using lines. Additionally, the small intestine is further divided into four regions: regions 9 and 10 represent the upper and lower portions of the jejunum, while regions 11 and 12 represent the upper and lower portions of the ileum. The size of the largest implant, known as the lesion size (LS), is assessed using a scale ranging from 0 to 3	LS-0 means that there are no plants visible in any of the areas. LS-1 refers to implants that are visible up to 0.5 cm in maximum diameter. LS-2 identifies nodules larger than 0.5 cm and up to 5 cm. LS-3 refers to implants with a diameter of 5 cm or greater. If an organ is extensively covered by tumor or if there are tissue adhesions, the region or site is also evaluated as LS-3. The lesion sizes are then summed for all abdominopelvic regions. A numerical score from 0 to 39 indicates the extent of the disease in all regions.

## Data Availability

The data are available upon request.
